# Computational modeling of cyclotides as antimicrobial agents against *Neisseria gonorrhoeae* PorB porin protein: integration of docking, immune, and molecular dynamics simulations

**DOI:** 10.3389/fchem.2024.1493165

**Published:** 2024-11-25

**Authors:** Muzamal Hussain, Nazia Kanwal, Alishba Jahangir, Nouman Ali, Nimra Hanif, Obaid Ullah

**Affiliations:** ^1^ Department of Biological Sciences, Faculty of Sciences, The Superior University, Lahore, Punjab, Pakistan; ^2^ The Institute of Physiology and Pharmacology, Faculty of Veterinary Science, The University of Agriculture, Faisalabad, Punjab, Pakistan; ^3^ Department of Biotechnology, Faculty of Science and Technology, University of Central Punjab, Lahore, Pakistan; ^4^ Department of Computer Science, Faculty of Sciences, University of Agriculture, Faisalabad, Punjab, Pakistan

**Keywords:** *Neisseria gonorrhoeae*, Porb porin, cyclotide, multi drug resistant (MDR), antibiotic resistance, insilico

## Abstract

**Background:**

*Neisseria gonorrhoeae* is the bacterium responsible for gonorrhoea, one of the most common sexually transmitted infections (STIs) globally. In 2020, the World Health Organization (WHO) estimated 82.4 million new cases of *Neisseria gonorrhoeae* infections. Current treatments rely on antibiotics, but the emergence of multi drug resistance (MDR) strains poses a significant threat to public health. This research aims to use computational modeling of cyclotides as antimicrobial agents targeting the *Neisseria gonorrhoeae* PorB Porin protein to inhibit its pathogenicity.

**Methodology:**

The PorB Porin protein was retrieved from the Protein Data Bank (PDB ID: 4AUI), cleaned, and visualized using Discovery Visual Studio. Physicochemical properties were predicted using ProtParam. Cyclotides were obtained from the CyBase database, with 3D models generated and refined via the Swiss Model for docking studies. HDOCK was used for molecular docking. Toxicity and allergenicity predictions were performed with ToxinPred and AlgPred. A heatmap of the peptide was created using Protein-Sol. Molecular dynamics (MD) simulations were conducted for 100,000 picoseconds using Desmond from Schrödinger LLC, while binding energy was analyzed using MMGBSA. Immune response simulations were done with C-ImmSim 10.1, and peptide simulation in water was performed via WebGro.

**Results:**

The protein’s GRAVY value is −0.539, indicating moderate hydrophilicity, and its isoelectric point is 9.14, suggesting a fundamental nature. Globa D had the highest docking score (−270.04 kcal/mol) and was deemed non-toxic and non-allergenic. MD simulations showed stable protein-ligand interactions, and MMGBSA revealed a low binding energy of −36.737 kcal/mol. Immune simulations indicated an effective immune response and peptide simulations demonstrated Globa D’s stability in water, making it a potential candidate for pharmaceutical applications.

**Conclusion:**

Globa D proved the best drug candidate against *Neisseria gonorrhoeae* by inhibiting PorB Porin protein chain A. Further *in vitro* and *in vivo* studies are recommended to validate these findings and explore clinical applications.

## 1 Introduction

*Neisseria gonorrhoeae* is the bacterium that infects humans, causing gonorrhoea, which is one of the most prevalent STIs worldwide. The WHO predicted 82.4 million new cases of *Neisseria gonorrhoeae* infection in individuals aged 15–49 in 2020. Homosexuals, transgender people and sex workers with a high prevalence of the disease are believed to be the most vulnerable to this infection (Nekahiwot and Debela, 2024). In women, if left untreated, gonorrhoea can cause apparent health problems such as pelvic inflammatory diseases, infertility, ectopic pregnancy and a raised risk of both contracting and spreading HIV (Ikokwu et al., 2023). The current treatment mainly entails antibiotics; however, the development of MDR strains of *N. gonorrhoea*e significantly threatens public health. Specifically, these resistant strains prevent the efficient control of current antibiotic therapeutic choices and prolong the attempts to manage the disease (Aitolo et al., 2021).

PorB Porin protein is one of the important determinants of virulence and resistance to *Neisseria gonorrhoeae* antibiotics. It helps the bacterium avoid being recognized and attacked by the host immune system, hence setting an abode for the infection. It acts as a path or a pore within the outer membrane, enabling the transportation of nutrients and waste products (Jones et al., 2024). It also interacts with host cell receptors to enable the bacteria to penetrate through the membrane and get into the advanced level of infection (Gao and van der Veen, 2020). PorB has been very much involved in the resistance to several antibiotics as it increases the impermeability of the bacterial cell envelope and denies access to many drugs (Sun et al., 2010). The structure is characterized by a positively charged channel organized by phosphate ions and ATP binding residues. Supporting substrate selectivity via hydrogen bond interactions and connecting Porin molecules to the peptidoglycan network, the narrowing of the barrel diameter is achieved by the β-bulge in the β2-strand and the lengthy L3 loop (Zeth et al., 2013). These protein characteristics make it essential to strategize new ways to combat the bacterium causing gonorrhoea.

Cyclotides are group of cyclic peptides from plants, are considered one of the most holistic candidates for new antimicrobial agents. These peptides have circular structures with multiple disulfide bonds, giving them extraordinary stability. Cyclotides have exhibited tremendous efficacy against infections caused by bacteria, fungi and viruses (de Veer et al., 2017). Their action mechanism mostly tends to damage bacterial membranes and subsequently cause cell bursting and death. Based on the impressive reversing capacity to superoxide anion, expression of antimicrobial solid activity, and structural rigidity, cyclotides can be of great significance for synthesizing new drugs for eliminating antibiotic-resistant microorganisms (Ojeda et al., 2019). Future research on the use of cyclotides in the treatment of gonorrhea is worthwhile, given the urgent need to develop new drugs due to the increasing emergence of drug resistance.

**FIGURE 1:**
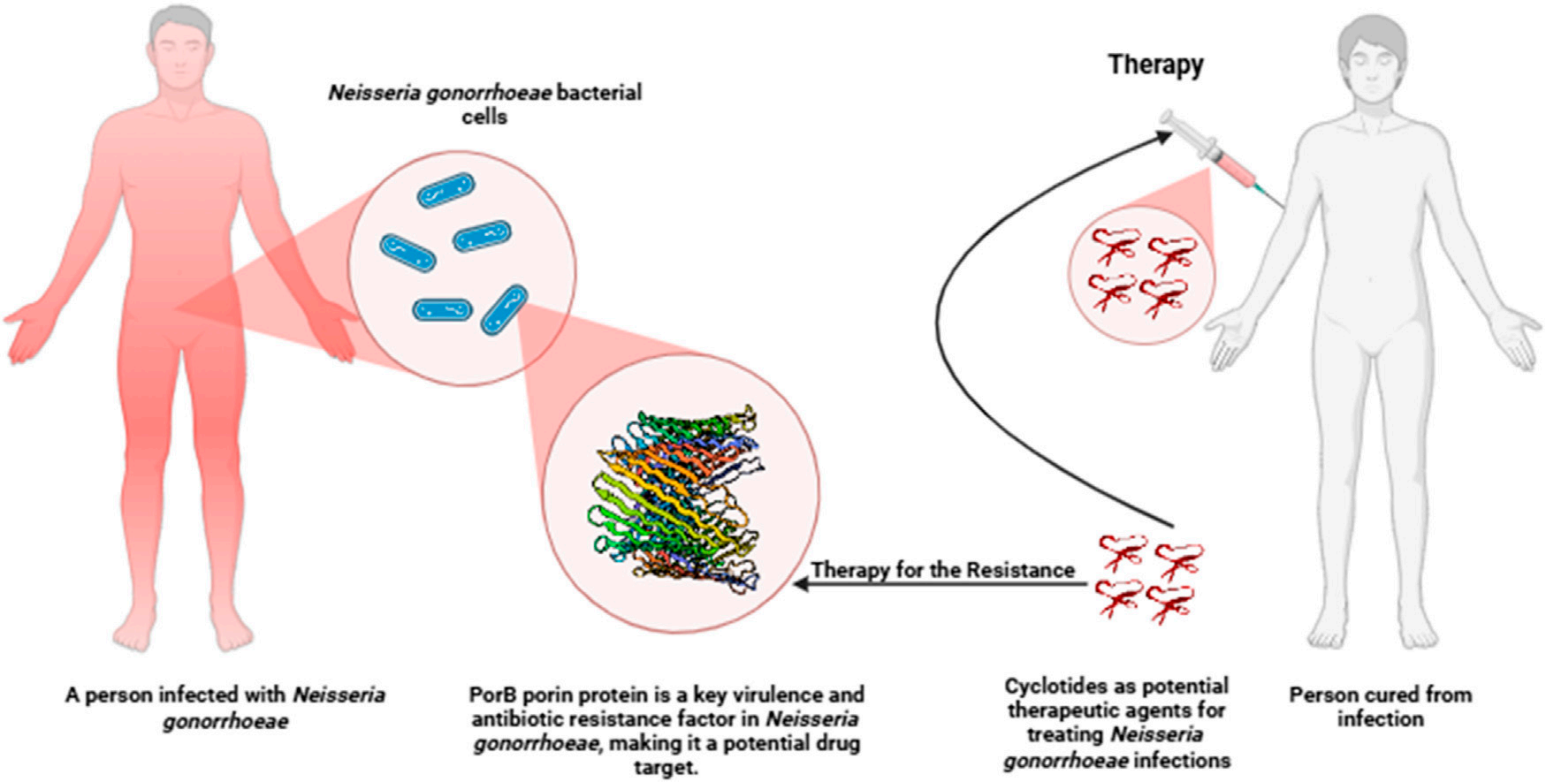
Schematic diagram of a potential therapeutic approach targeting PorB protein in *Neisseria gonorrhoeae* infection using cyclotides.

**FIGURE 2:**
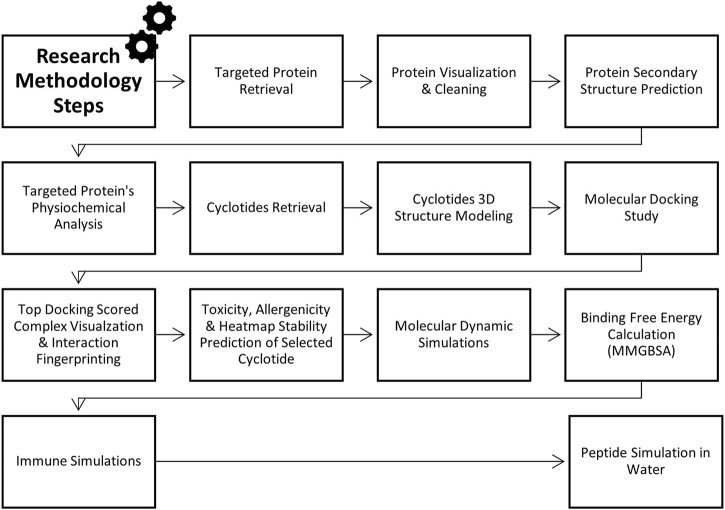
Methodology flowchart.

This research employs the insilico approaches to design peptide-based drugs derived from cyclotides that selectively target the PorB Porin protein of *Neisseria gonorrhoea* (Figure 1). Therefore, molecular docking and dynamic simulations were employed to identify and predict cyclotide-derived peptides that could accurately treat the PorB Porin protein. Hence, it will be essential to offer this study as a part of filling the lack of the corresponding element within current management techniques and as the creation of a new specific approach to the eradication of *N. gonorrhoea*. The incorporation of insilico approaches aids in the increase in the rate of the drug discovery process. It is the best approach to producing potent antimicrobial compounds mainly because this pathogen is gradually developing drug resistance. The methodology flowchart can be seen in Figure 2.

## 2 Methodology

### 2.1 Targeted protein retrieval and structure analysis

The PorB Porin protein of *Neisseria gonorrhoeae* was obtained from the Protein Data Bank (PDB) (http://www.rcsb.org) with the ID 4AUI having resolution 3.20 Å (Zeth et al., 2013). Since the PDB provides information on the protein structure at the atomic level, it can be used for accurate modelling. The structure was saved in. pdb format, which is suitable for updating the input for subsequent computational analysis (Burley et al., 2023). The protein structure was cleaned and visualized using Biovia Discovery Visual Studio (https://discover.3ds.com/discovery-studio-visualizer-download) to remove extra ligands and water molecules (Baroroh et al., 2023). The PSIPRED tool (http://bioinf.cs.ucl.ac.uk/psipred) was part of this computational procedure, which provided a comprehensive study of the protein fold’s secondary structures. The result was taken in the PIPRED chart cartoon (McGuffin et al., 2000). By using SOPMA (https://npsa-pbil.ibcp.fr/NPSA/npsa_sopma.html), the quantity of alpha helices, extended strands, and random coils was anticipated (Geourjon and Deleage, 1995).

### 2.2 Physiochemical properties analysis of protein

The ProtParam tool on the ExPASy server (https://web.expasy.org/protparam/) was used to predict the protein’s physicochemical properties Table 1 provides relevant information on the protonation states of protein residues. This tool predicts the protein’s molecular weight, theoretical isoelectric point, amino acid composition, extinction coefficient, and instability index from the given protein sequence. The estimated properties were then compared to determine the protein’s stability and activity in various conditions (Bidkar et al., 2014).

**TABLE 1:** Physiochemical properties of toxin protein PorB porin.

Physiochemical properties of toxin protein PorB porin		
Characteristics	Properties	Values (globa D)
Atomic and Amino-Acid Composition	Number of Amino Acids	327
	Total Number of Atoms	4,966
	Formula	C_1592_ H_2430_ N_456_ O_486_ S_2_
	Molecular Weight	35,797.69
State of Charge on Residues	Negatively Charged Residues (Asp + Glu)	32
	Positively Charged Residues (Arg + Lys)	38
Extinction Coefficients	(M^-1^ cm^-1^ at 280 nm) Measured in Water	Ext. Coefficient: 54,320 Abs 0.1% (=1 g/L) 1.517
Estimated Half-Life	Mammalian reticulocytes, *In-Vitro*	30 h
	Yeast, *In-Vivo*	>20 min
	*Escherichia coli*, *In-Vivo*	>10 h
Protein Stability Indices	Aliphatic Index	67.40
	Instability Index	30.72 (Stable Protein)
Protein Hydrophobicity and Isoelectric Parameters	GRAVY (Grand Average of Hydropathicity)	−0.539
	Theoretical pI	9.14

### 2.3 Cyclotides retrieval and 3D structure prediction

Cyclotides were retrieved from the CyBase database (http://research.imb.uq.edu.au/cybase) in FASTA format, and further information on the sequences and structures was obtained. CyBase is an informative and integrated database and tool for cyclic peptides, which contains the series, configuration and activities of cyclic peptides (Wang et al., 2007). The three-dimensional models were generated using the Swiss Model and refined for docking studies. SWISS-MODEL (https://swissmodel.expasy.org) is a web-based tool that provides a fully automated service for the homology modelling of protein structures for structural bioinformatics and molecular biology (Schwede et al., 2003).

### 2.4 Docking analysis

Docking analysis was performed using the HDOCK tool (http://hdock.phys.hust.edu.cn/). The cleaned PDB file of the PorB protein (with ligands and water removed) was uploaded as the receptor, with Chain A selected. Cyclotides were uploaded as ligands, and blind docking was performed without specifying advanced options like template-free docking or symmetric multimer docking. This approach enabled unbiased interaction analysis across Chain A of PorB. HDOCK is a comprehensive web software for protein-protein and protein-DNA/RNA interface docking, which includes both template-based modelling and complimentary docking for estimating the structure of the complex. The results were analyzed to identify the most favorable binding interactions and their potential biological relevance (Yan et al., 2017). Docking visualization of top scored complex and binding site identification was performed by using PyMol (https://www.pymol.org/) (Rauf et al., 2015). PDBsum online tool was used to interpret detailed analysis of docking interaction (Laskowski et al., 2018) and DIMPLOT in LIGPLOT was employed to validate the docking interaction analysis (Savar and Bouzari, 2014).

### 2.5 Toxicity, allergenicity and heatmap stability prediction of selected cyclotide

The top cyclotides’ toxicity and allergenicity were predicted using the ToxinPred (https://webs.iiitd.edu.in/raghava/toxinpred/design.php) and AlgPred (https://webs.iiitd.edu.in/raghava/algpred/submission.html) online tools. ToxinPred utilized the amino acid sequences to predict the toxic effects (Sharma et al., 2022), while AlgPred used the sequences to predict allergenicity (Sharma et al., 2021). These evaluations are critical to ascertain the safety and efficacy of cyclotides as therapeutic agents. Protein-Sol server (https://protein-sol.manchester.ac.uk/heatmap) was used to determine the heatmap of peptide stability. The results were obtained in the form of an energy heatmap graph and a charge heatmap graph [(Hebditch and Warwicker, 2019; Hebditch et al., 2017)].

### 2.6 Molecular dynamic simulations, PCA and DCCM analysis

MD simulations were performed for 100,000 picoseconds using Desmond from Schrödinger LLC. Initial protein-ligand complexes were obtained from docking studies, which provide static binding predictions. MD simulations, using Newton’s classical equations, predict atomic movements over time in physiological environments. Complexes were preprocessed, optimized, and minimized using the Protein Preparation Wizard in Maestro. An orthorhombic simulation box was created to house the system, maintaining a minimum distance of 10 Å between the protein and the box edges to prevent interactions with periodic images. The system was prepared with the System Builder tool, employing the TIP3P solvent model, the OPLS_2005 force field, and including 0.15 M sodium chloride to mimic physiological conditions. The system was solvated with approximately 15,000 TIP3P water molecules, ensuring a suitable hydration layer around the protein. The NPT ensemble was utilized, with temperature regulated at 300 K using the Langevin thermostat and pressure maintained at 1 atm with the Martyna-Tobias-Klein barostat. Trajectories were saved every 100 ps, enabling detailed analysis of the system dynamics over time. Stability and post simulation results was evaluated by calculating the RMSD, RMSF, Radius of Gyration, and Hydrogen Bonds of the protein and ligand over time (Shivakumar et al., 2010; Ahammad et al., 2021). The principal component analysis (PCA) and dynamic cross-correlation matrix (DCCM) were also evaluated by applying the R package “Bio3D” (Grant et al., 2021).

### 2.7 MM-GBSA energy prediction

The MM-GBSA analysis was performed using the OPLS 2005 parameters in Prime (Schrödinger Suite). The binding energy, denoted as ΔG_bind, was calculated by subtracting the free ligand and receptor energies from the optimized complex’s Prime energy. This analysis also evaluated various energy contributions, including ΔG Bind Coulomb, ΔG Bind Lipo, ΔG Bind H bond, ΔG Bind Solv GB, ΔG Bind Packing, ΔG Bind vdW, and ΔG bind SelfCont. Strain energies were assessed by contrasting the receptor and ligand geometries in their free and optimized complex forms. The analysis allowed for energy breakdown, ensuring that all parameters had no missing side chains. A comprehensive analysis of the entire MD trajectory was conducted to enhance the accuracy of the MM-GBSA binding energy calculations (Chinnasamy et al., 2020a).

### 2.8 Immune simulations of docked complex

The immune response to a cyclotide-targeted protein complex was studied by simulating its interactions using the C-ImmSim server (https://kraken.iac.rm.cnr.it/C-IMMSIM/index.php). Cytokines, interferons, and antibody production were assessed using the PSSM technique (Rapin et al., 2010). Predictions on the immunological responses of T helper cells were also made using the default parameters assigned by the server to quantify immune variety, which the Simps Index represents.

### 2.9 Peptide simulation in water

A simulation of cyclotide in water was carefully planned and executed to determine the possibility of cyclotide as a liquid or syrup-based drug. This simulation was performed by using WebGro server (https://simlab.uams.edu/ProteinInWater/index.html) (Prasetyo et al., 2023). The simulation process was meticulously designed to make it as realistic as possible. First, the protein structure was cleaned up to remove any errors and then the protein structure was energy minimized for 5,000 steps using the steepest descent method. The system was equilibrated under the NVT/NPT ensemble at 300 K and 1.0 bar pressure to maintain the system. The molecular dynamics simulation was then performed for 50 ns using the leap-frog integrator, with the GROMOS96 43a1 force field and SPC water model. The system was neutralized and maintained at 0.15 M NaCl concentration within a triclinic box type (Gunasekaran et al., 2023).

## 3 Results

### 3.1 Target protein physiochemical properties analysis

**FIGURE 3:**
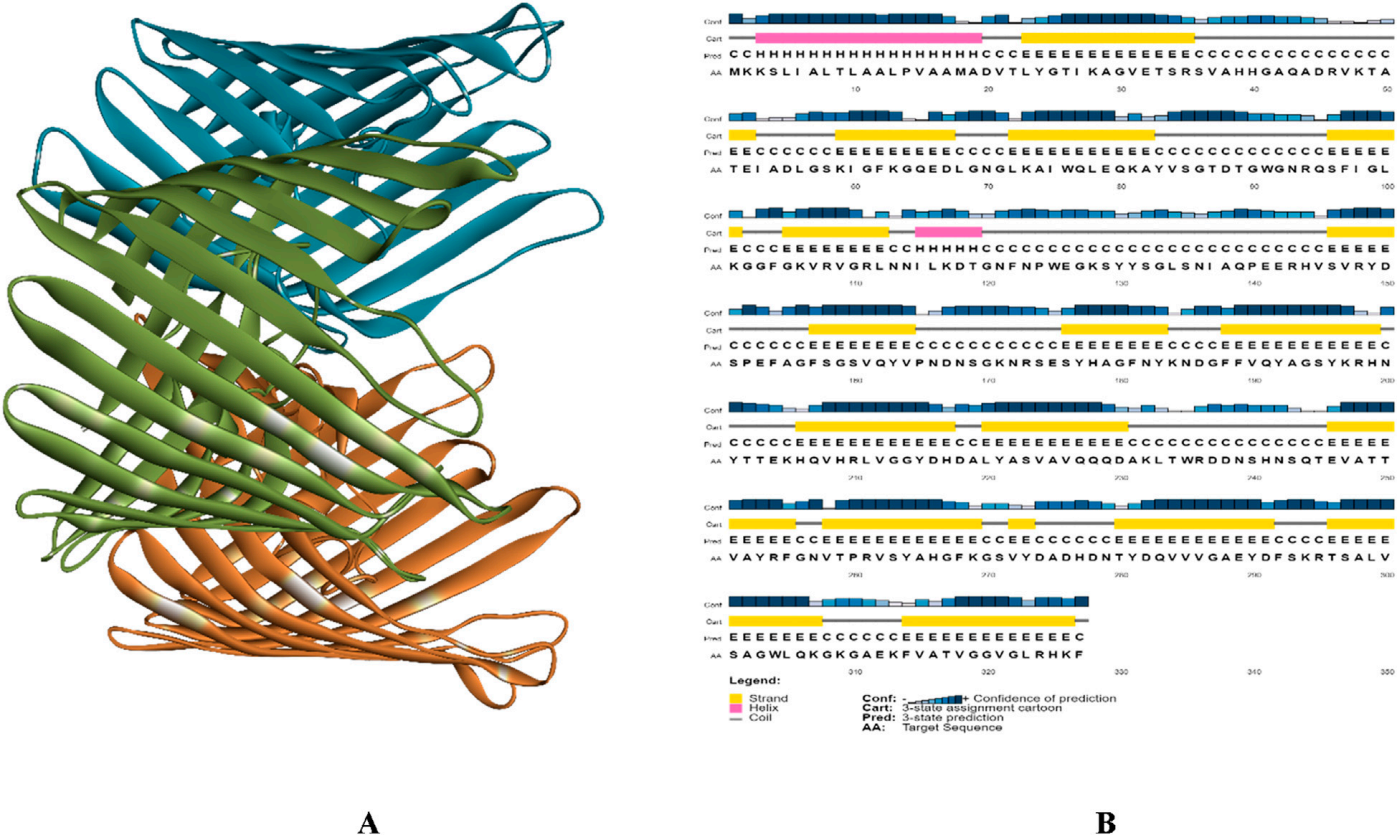
**(A)** 3D structure of PorB Porin protein of *Neisseria gonorrhoeae,* Chain A presented in green color Chain B presented with blue and Chain C with orange color, and **(B)** PSIPRED chart cartoon of analysis of secondary structure of PorB Porin protein from *Neisseria gonorrhoeae*.

Figure 3A illustrates the structure of PorB Porin protein of *Neisseria gonorrhoeae* retrieved from PDB. It possesses three chains Chain A, Chain B, and Chain C. SOPMA revealed that PorB Porin have 13.76% (45 residue) of alpha helices, 27.52% (90 residue) extended strands and 58.72% (192 residues) of random coils. Figure 3B shows the PSIPRED chart cartoon indicating component of secondary structure of protein. The GRAVY value of protein is −0.539 indicate its moderately hydrophilic nature. The theoretical pI (isoelectric point) value is predicted to be 9.14 which indicates the basic nature of protein. Table 1 indicates the physiochemical properties of targeted protein.

### 3.2 Docking analysis results

The complex formed between the cyclotide and the target protein was predicted using the HDOCK server, and the information was organized as a table. Table 2 assisted in a straightforward comparison of the docking outcomes between various cyclotides and targeted protein, and the top docking score complex was used for further analysis.

**TABLE 2:** Cyclotide and PorB porin docking results.

Cyclotide and PorB porin docking results	
Cyclotides	Docking score
Viphi A	−232.79
Viphi B	−223.66
Viphi C	−234.34
Viphi D	−245.11
Viphi E	−252.23
Viphi F	−244.30
Viphi G	−250.98
Cycloviolacin O13	−251.97
Cycloviolacin O14	−238.42
Cycloviolacin O18	−237.17
Cycloviolacin O20	−232.63
Cycloviolacin O23	−260.86
Cycloviolacin O24	−240.32
Cycloviolacin O25	−266.90
Vibe 1	−231.14
Vibe 2	−228.57
Vibe 3	−253.51
Vibe 6	−223.85
Vibe 7	−223.63
Vibe 12	−265.07
Vibe 16	−235.69
Vibe 18	−226.37
Vibe 19	−241.61
Vibe 20	−233.76
Vibe 21	−262.69
Vibe 23	−230.31
Vibe 24	−243.98
Vibe 25	−252.32
Glopa A	−228.26
Glopa B	−226.62
Glopa C	−246.29
Glopa D	−220.24
Glopa E	−226.30
Glopa F	−262.24
Glopa G	−258.29
Globa A	−238.78
Globa B	−259.72
Globa C	−243.86
Globa D	−270.04
Globa E	−219.69
Globa F	−236.91

### 3.3 Interaction analysis and visualization of top docking result

**FIGURE 4:**
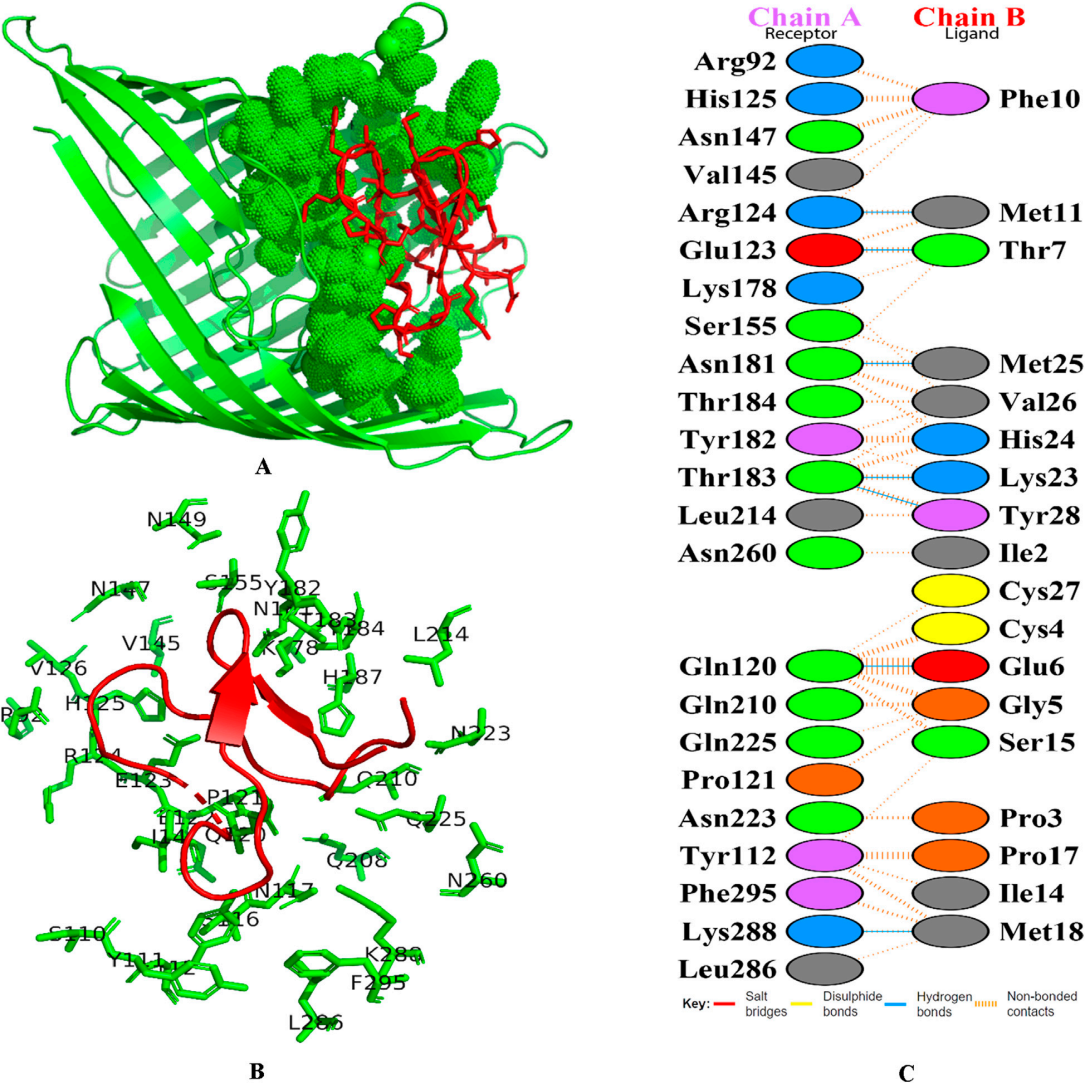
3D docking visualization of chain A of PorB Porin with cyclotide Globa D. **(A)** Image shows the protein’s molecular surface in green, with the active site shown in green spheres. Red sticks refer to the bound ligand area within the protein’s binding pocket. This map emphasizes the ligand’s spatial layout in regard to the protein’s active site, applying shading to areas of interaction and/or binding, and **(B)** Structural map of the protein with emphasis on the active site and the binding pocket. The green lines refer to the targeted protein’s amino acid residues involved in binding interactions, and the labels around them refer to specific amino acid positions and red color cartoon refers to the ligand, and **(C)** Figure shows the docking interaction analysis PDBsum. Chain A refers to receptor (Porb Porin) and chain B refers to ligand (Globa D).

Globa D was chosen as the best drug candidate because it represented the complex with the top docking score (−270.04) among all the complexes yielded by docking calculations. Figure 4 illustrated 3D docking and interaction visualization of Chain A of PorB Porin with Globa D and binding site visualization.

### 3.4 Results of toxicity, allergenicity and heatmap prediction of selected cyclotide

**FIGURE 5:**
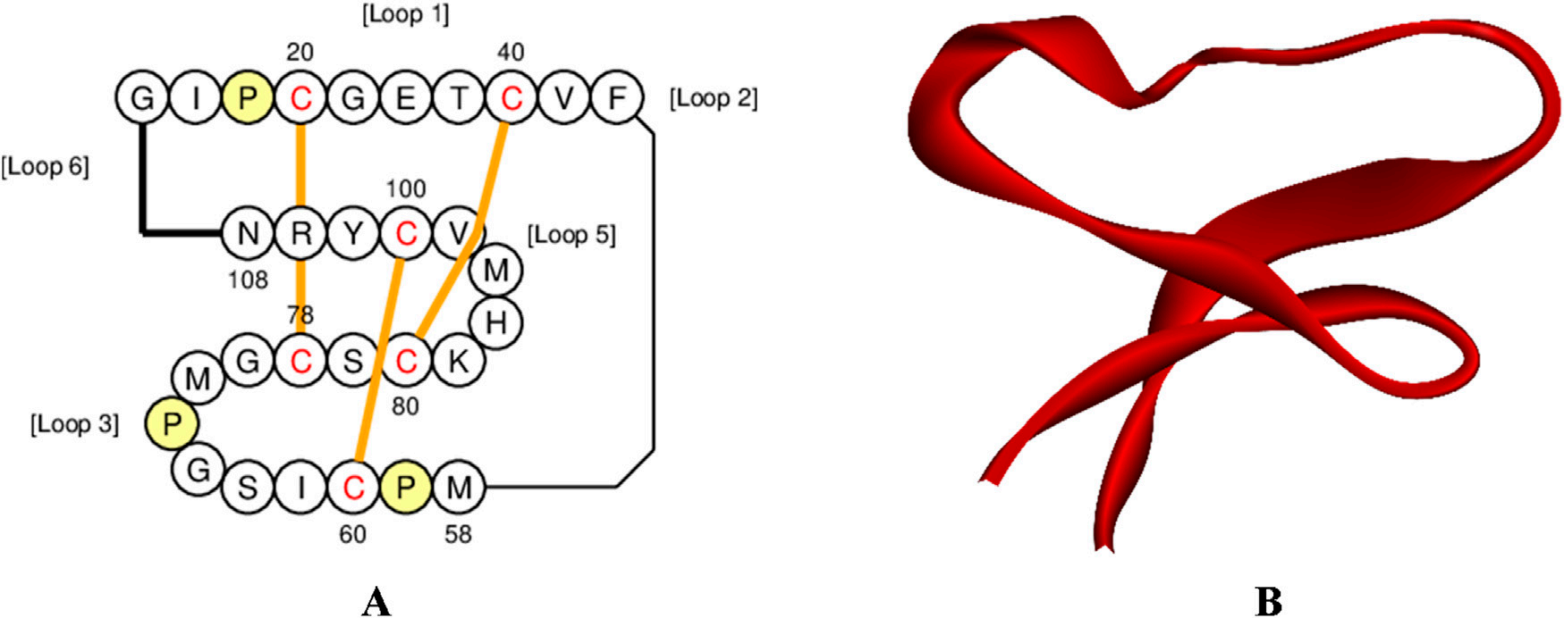
Structure visualization of Cyclotide Globa D **(A)** 2D structure of Globa D, and **(B)** 3D structure of Globa D.

**FIGURE 6:**
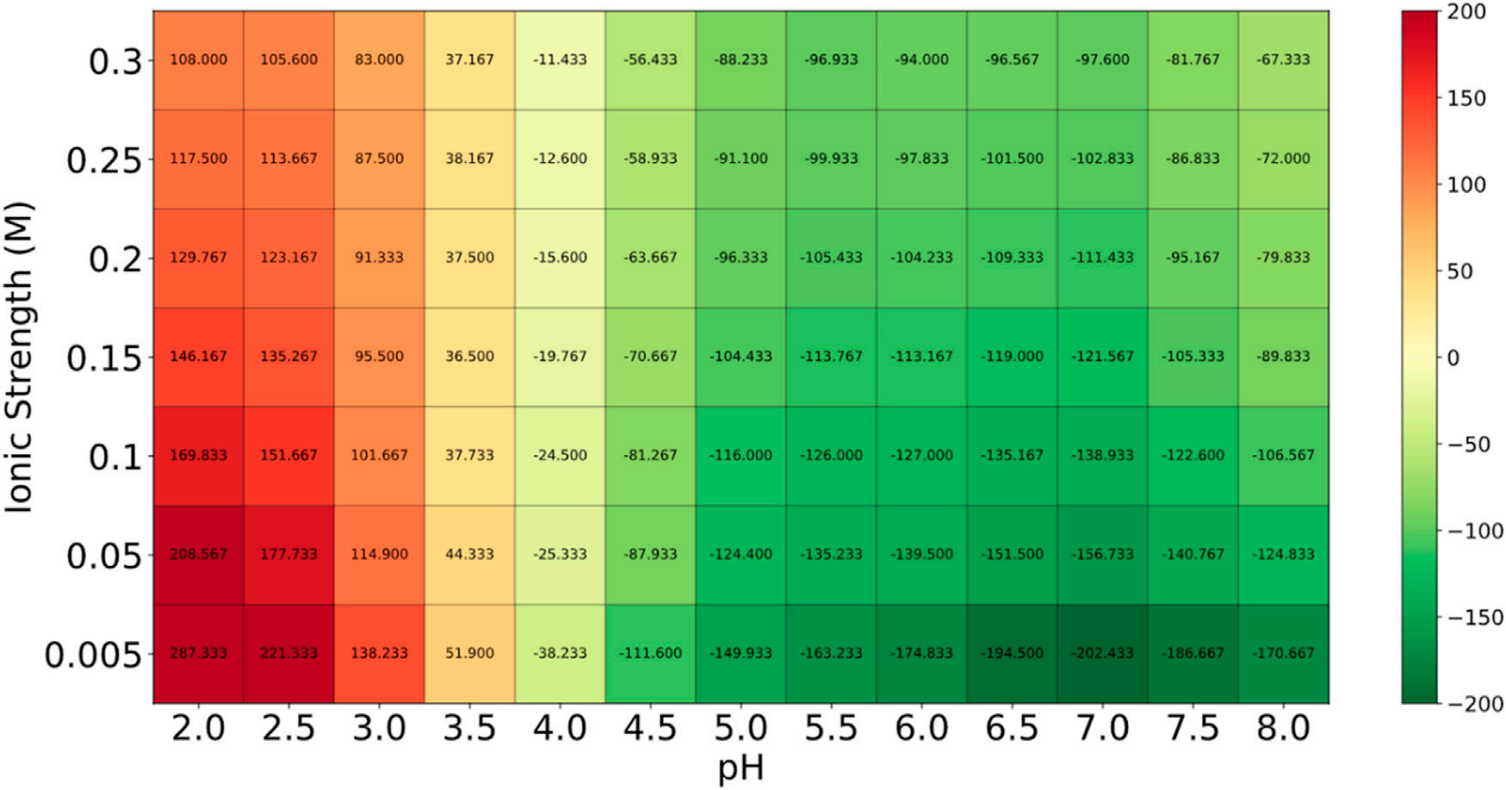
The energy values in the “Energy Heatmap” are presented in Joules per amino acid (J per aa). This heatmap illustrates how the peptide’s energy levels vary under different conditions. In this representation, green areas indicate higher stability (lower energy), while red areas signify lower stability (higher energy). The peptide exhibits optimal stability in the green regions, suggesting that these conditions are favorable for its potential use as a drug.

**FIGURE 7:**
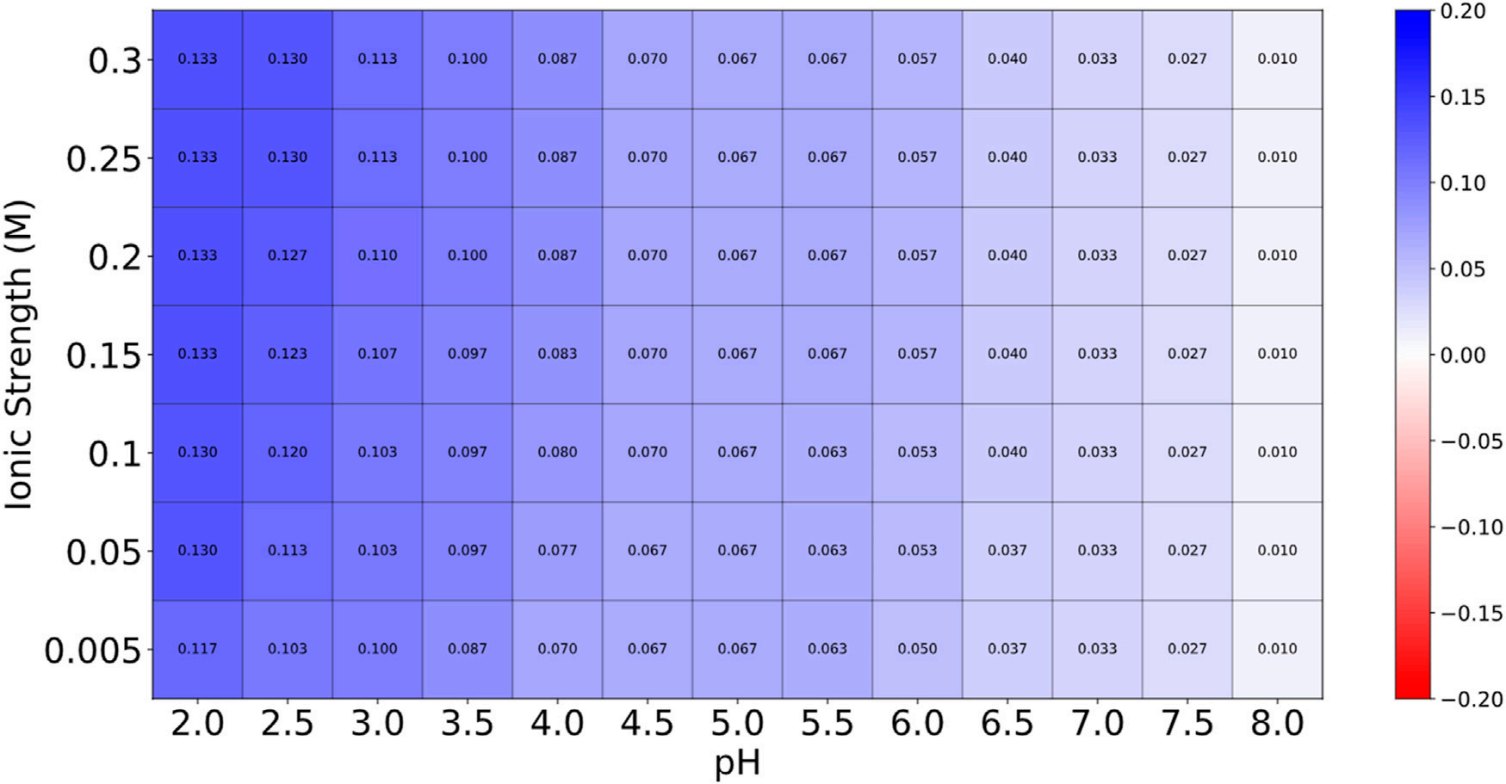
The values in the “Charge Heatmap” are expressed in electrons per amino acid (e per aa). This heatmap provides insights into the peptide’s charge behavior under various conditions. Low charge density is represented by blue, while high charge density is indicated by red. The predominance of blue across the heatmap suggests that the peptide maintains a relatively stable charge across different conditions, which is advantageous for its stability. The limited red areas indicate that only a few conditions exhibit higher charge densities, but these instances are not predominant.

Globa D was chosen as the potential drug candidate due to top docking score (−270.04) among all the complexes. Table 3 presents the status of mutation, toxicity, and allergenicity of Globa D. Figure 5 shows 2D and 3D structure of Globa D. Table 4 shows the physiochemical properties of Globa D. Figure 6 illustrates the energy heatmap and Figure 7 shows the charge heatmap of Globa D.

**TABLE 3:** The presence of Mutation, toxicity, and allergenicity in Globa D.

Peptide	Mutation position	SVM score	Toxicity	Allergenicity
Globa D	No Mutation	−0.37	Non-Toxin	Non-Allergen

**TABLE 4:** Physiochemical properties of globa D.

Physiochemical properties of top cyclotide based on docking score		
Characteristics	Properties	Values (globa D)
Atomic and Amino-Acid Composition	Number of Amino Acids	30
	Total Number of Atoms	428
	Formula	C_133_ H_211_ N_37_ O_38_ S_9_
	Molecular Weight	3,224.90
State of Charge on Residues	Negatively Charged Residues (Asp + Glu)	1
	Positively Charged Residues (Arg + Lys)	2
Extinction Coefficients (M^-1^ cm^-1^ at 280 nm) Measured in Water	Assuming all pairs of Cys residues form cystines	Ext. Coefficient: 1865 Abs 0.1% (=1 g/1): 0.578
	Assuming all Cys residues are reduced	Ext. Coefficient: 1,490 Abs 0.1% (=1 g/1): 0.462
Estimated Half-Life	Mammalian reticulocytes, *In-Vitro*	30 h
	Yeast, *In-Vivo*	>20 min
	*Escherichia coli*, *In-Vivo*	>10 h
Protein Stability Indices	Aliphatic Index	45.33
	Instability Index	17.30 (Stable Protein)
Protein Hydrophobicity and Isoelectric Parameters	GRAVY (Grand Average of Hydropathicity)	0.410
	Theoretical pI	7.79

### 3.5 Molecular dynamic simulations, PCA and DCCM results

**FIGURE 8:**
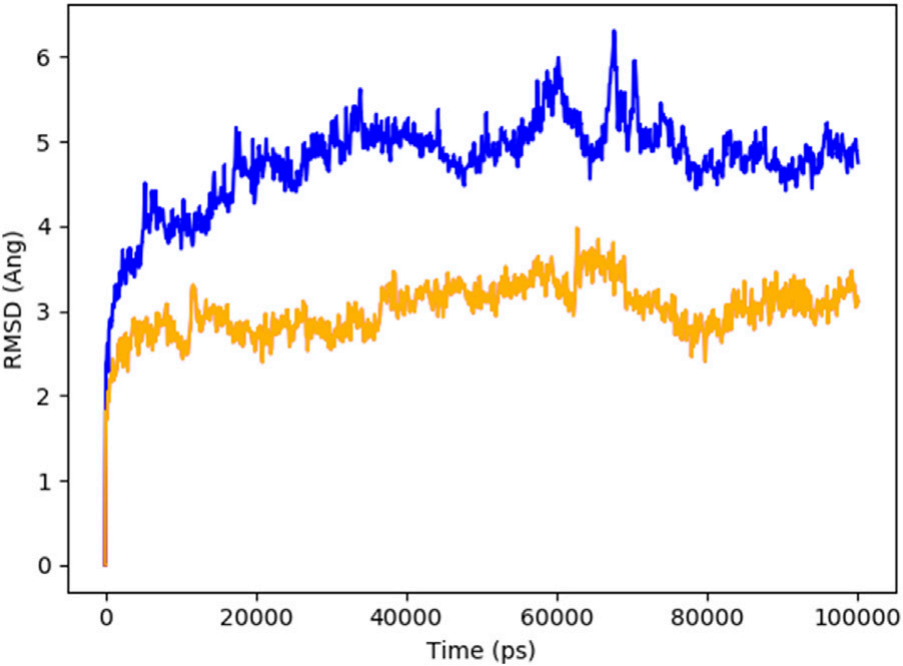
RMSD of PorB Porin (blue) and Globa D (orange) during the simulation time showed receptor flexibility and ligand stability.

**FIGURE 9:**
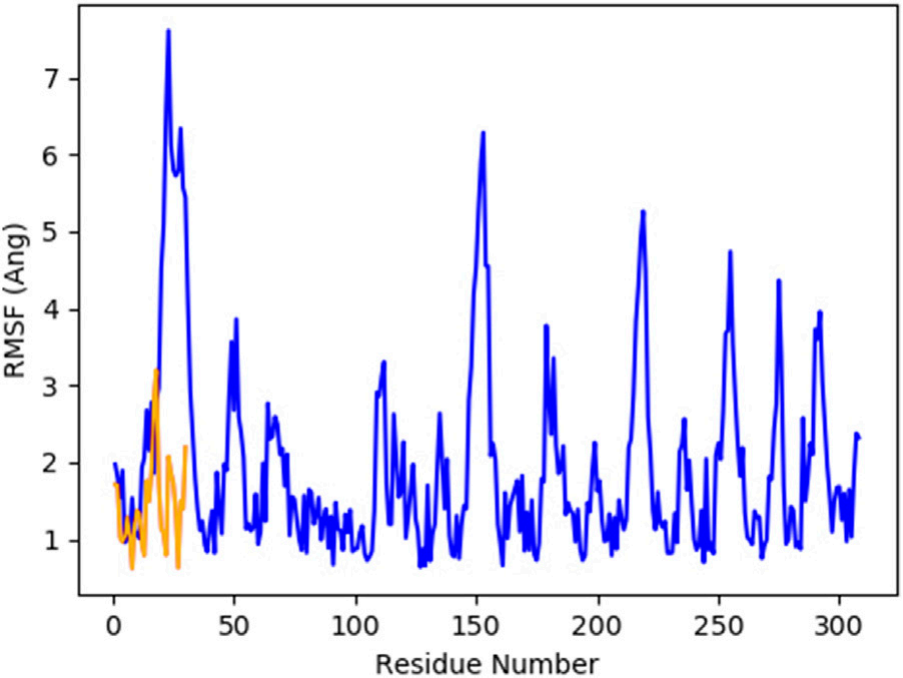
RMSF of PorB Porin (blue) and Globa D (orange) during the simulation time showed receptor flexibility and ligand stability.

**FIGURE 10:**
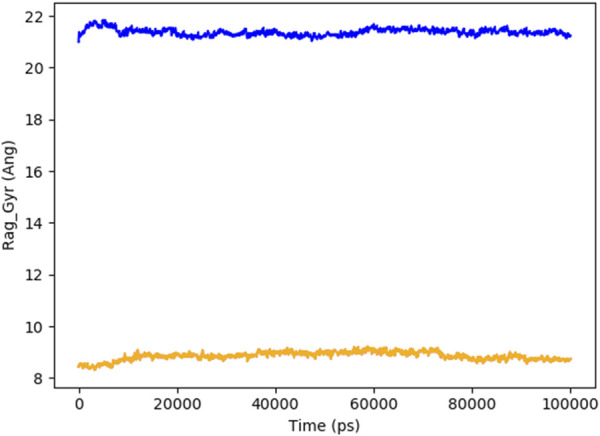
The radius of gyration of PorB Porin (blue) and Globa D (orange) with time shows that the receptor is more extended, and the ligand is more compact.

**FIGURE 11:**
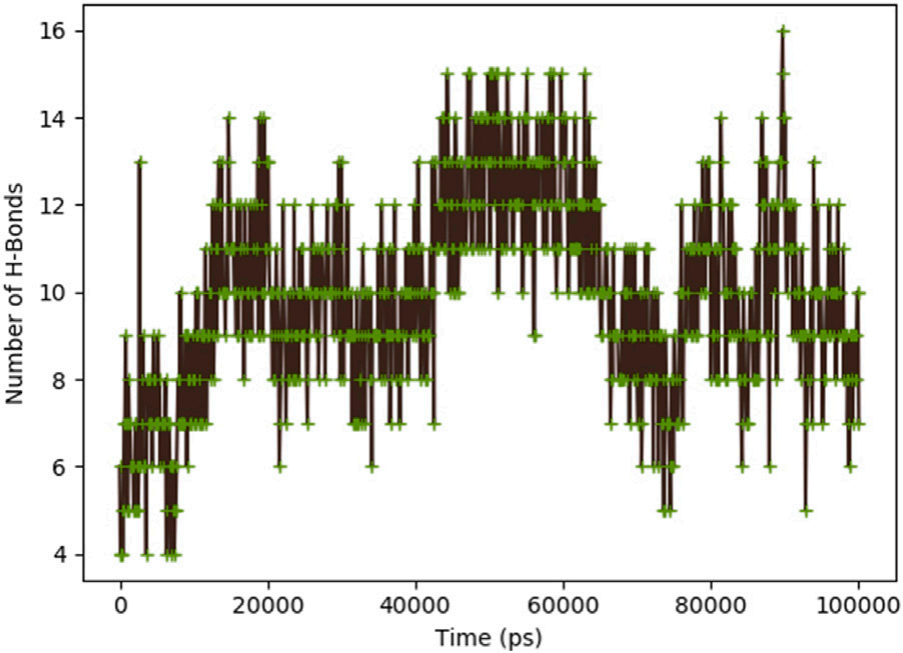
Dynamic binding interactions between PorB Porin and Globa D over time, regarding the number of hydrogen bonds.

**FIGURE 12:**
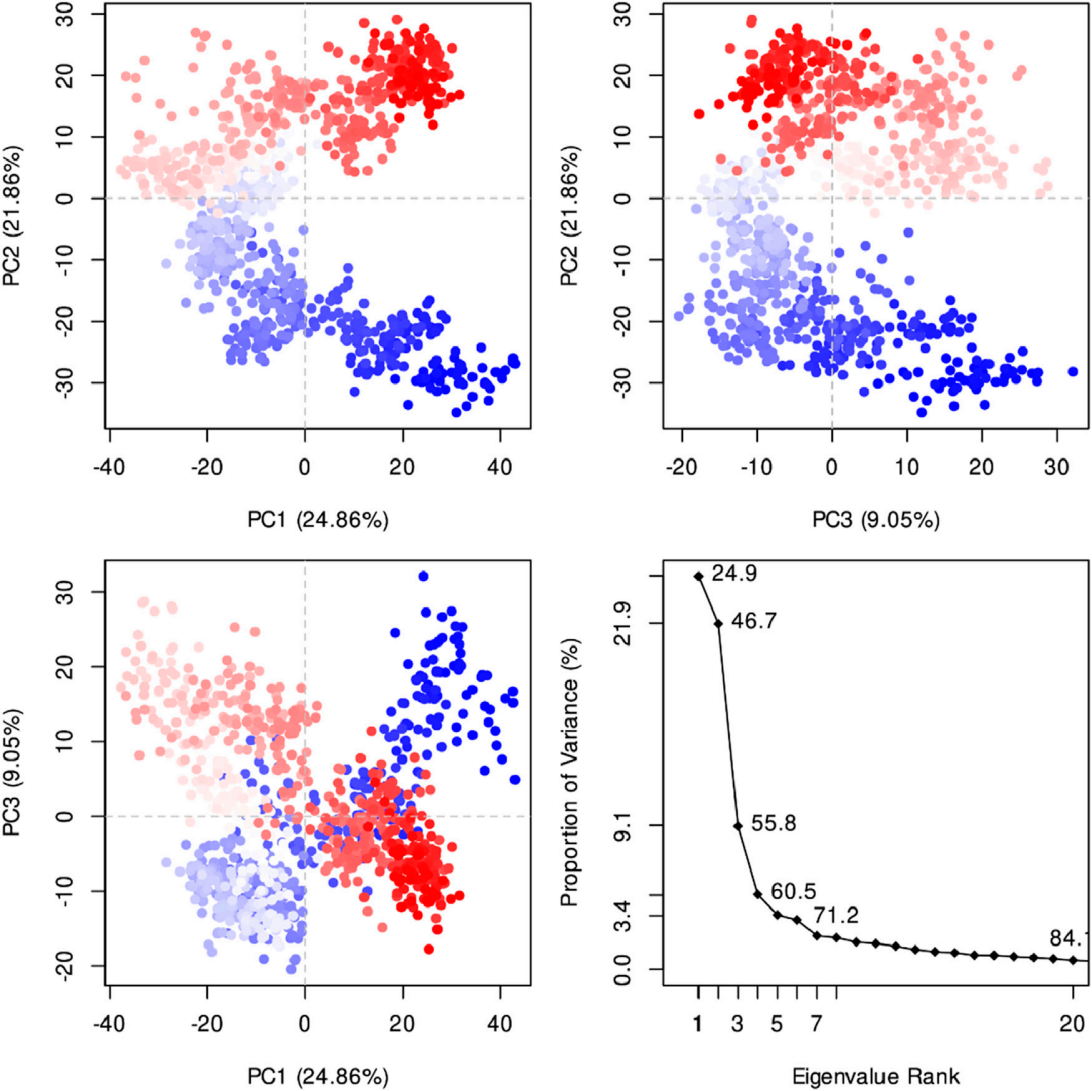
The PCA diagram revealed that the first three panels show the clustering of data points along PC1 vs. PC2 (24.86% vs. 21.86% variance explained), PC2 vs. PC3 (21.86% vs. 9.05% variance explained), and PC1 vs. PC3 (24.86% vs. 9.05% variance explained), respectively, illustrating the separation of distinct conformations. The fourth panel displays the scree plot, showing the proportion of variance explained by each principal component, with the first few components capturing the most significant variation. The first principal component (PC1) explains 24.9% of the variance, followed by 21.9% for PC2, 9.1% for PC3, and progressively lower values for subsequent components.

**FIGURE 13:**
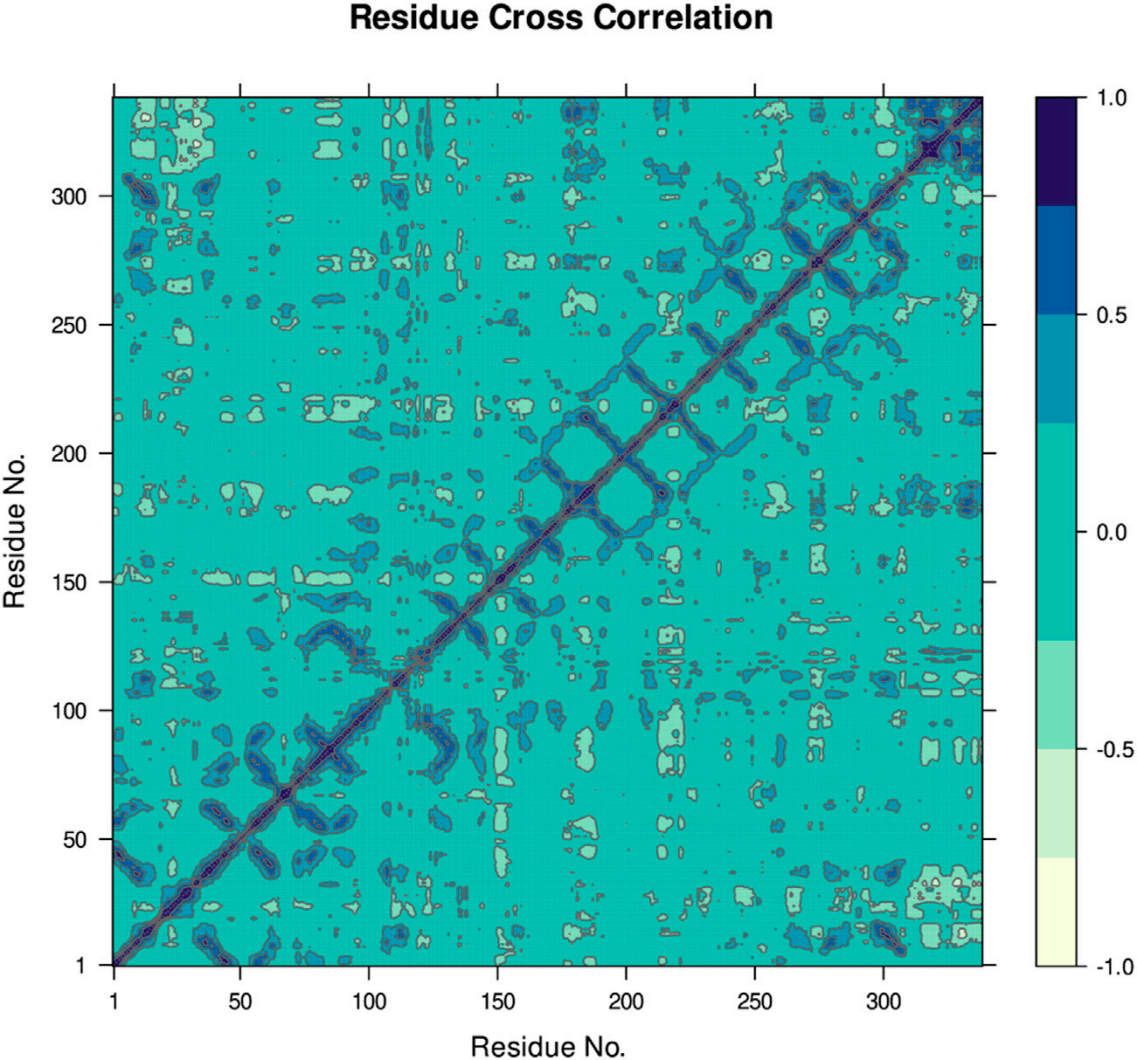
The DCCM provides the dynamic correlation between the residues in an organism’s protein structure. In this case, positive correlations are dark blue and indicate interacting movements, while negative correlations are lighter shades of blue and show that the movements are counteractive.

Figure 8 indicates the RMSD of Receptor (PorB Porin in blue) and ligand (Globa D in orange). The receptor has high RMSD values (∼5 Å) and a high level of flexibility. The ligand has lower and stable RMSD values (∼2–3 Å), which suggests that the ligand’s conformation is stable. Figure 9 illustrates the RMSF. The receptor has high fluctuations at some of the residues (up ∼ to 7 Å), which represent the flexible regions. The ligand has much lower RMSF values, which means that the ligand is well bound to the receptor. The radius of gyration in Figure 10 shows the receptor has a high radius of gyration (∼22 Å), which is stable but extended conformation. The ligand has a lower and stable radius of gyration (∼8–9 Å), indicating that the ligand’s structure is compact and stable. Figure 11 shows the number of hydrogen bonds, revealing a consistently high level of interactions between the receptor and ligand, with a peak of approximately 15 bonds. This indicates a stable receptor-ligand interaction, maintained for 42.14% of the trajectory, underscoring the stability and robustness of the complex throughout the simulation period. Figure 12 illustrates the PCA diagram of complex and Figure 13 shows DCCM graph that provides the dynamic correlation between the residues in an organism’s protein structure.

### 3.6 MM-GBSA energy

Table 5 indicates MM-GBSA results suggesting that binding occurs mainly through lipophilic and van der Waals forces. The total binding free energy of −36.7376 kcal/mol is considered favorable, and it is understood that the ligand has moderate binding to the target. The Coulombic energy is relatively positive than other predicted components of binding energy, but the other components, particularly vdW, Hbonds, and Lipo energies, have the lowest negative energy, suggesting stable interactions.

**TABLE 5:** Calculation of the average MM-GBSA binding energy of Globa D with PorB Porin.

MM-GBSA energy results	
Energies (kcal/mol)	Compound (PorB Porin-Globa)
Δ*G*_bind_	−36.7376
Δ*G*_bind_ Coulomb	105.0553
Δ*G*_bind_ H_bond_	−1.5054
Δ*G*_bind_ Lipo	−50.0930
Δ*G*_bind_ Packing	−3.1471
Δ*G*_bind_ SelfCont	−0.6386
Δ*G*_bind_ Solv-GB	−13.0728
Δ*G*_bind_ vdW	−95.8401

### 3.7 Immune simulations

**FIGURE 14:**
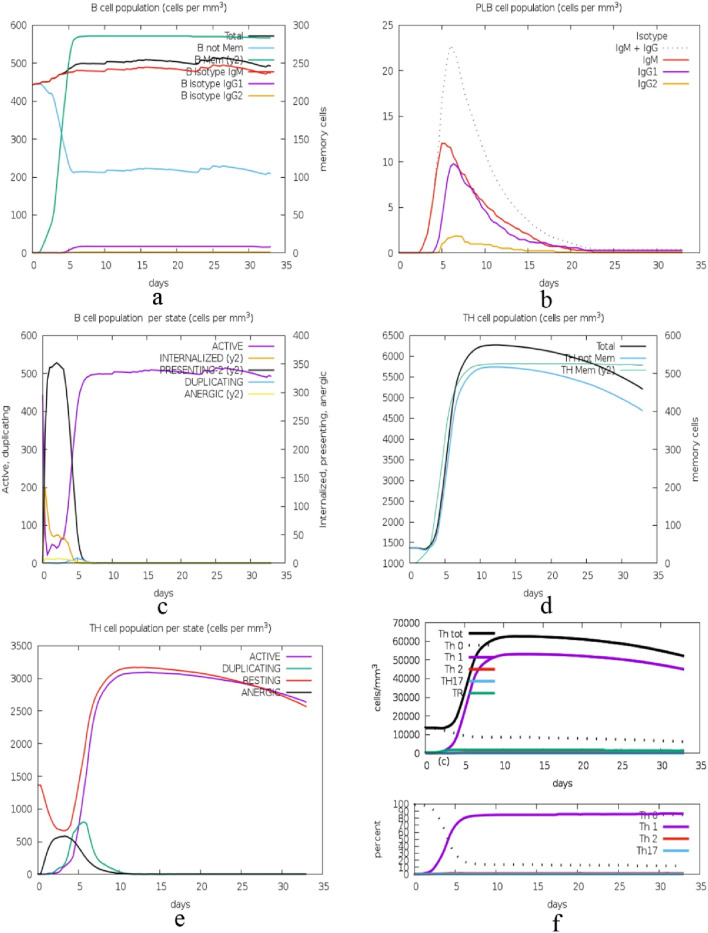
Immune cell population dynamics: this figure displays the time course of B and TH cell populations, and their subtypes and states, during a 35-day long immune response simulation, with the general pattern of increase followed by plateau, which is suggestive of adaptive immunity. **(A)**: Dynamics of total B cell populations, memory cells (B not Mem), and isotypes (IgM, IgG1, IgG2), showing an initial increase followed by stabilization, **(B)** Kinetics of plasma B cells (PLB) producing isotypes, with IgM appearing first and at the highest levels, **(C)** B cell activity states, where active and internalized states are short-lived, transitioning to anergic or resting states, **(D)** Total and memory helper T (TH) cell populations showing sharp increases and stabilization, similar to B cells, **(E)** TH cell states transitioning from active to resting or anergic states, and **(F)** Expansion and stabilization of various TH cell subtypes, including Th0, Th1, Th2, Th17, and Treg.

The immune simulation presented in Figure 14 illustrates the time-dependent changes in the numbers of different immune cells. In graph (a), the total B cell population and its subtypes, memory cells (B not Mem) and different isotypes (IgM, IgG1, IgG2) are increasing and then become stable. Figure b shows the kinetics of plasma B cells (PLB) producing different isotypes with IgM being the first and highest. Graph (c) illustrates the B cell activity states with active and internalized states as being short-lived and can either become anergic or resting. Graph (d) presents the total and memory helper T (TH) cell populations, which also rise sharply and then level off, as in the case of B cells. Graph (e) looks at TH cell states and there is a transition from the active state to the resting or anergic state. Lastly, graph f showing two graphs that depict the various TH cell subtypes including Th0, Th1, Th2, Th17 and Treg where they have expanded and stabilized.

**FIGURE 15:**
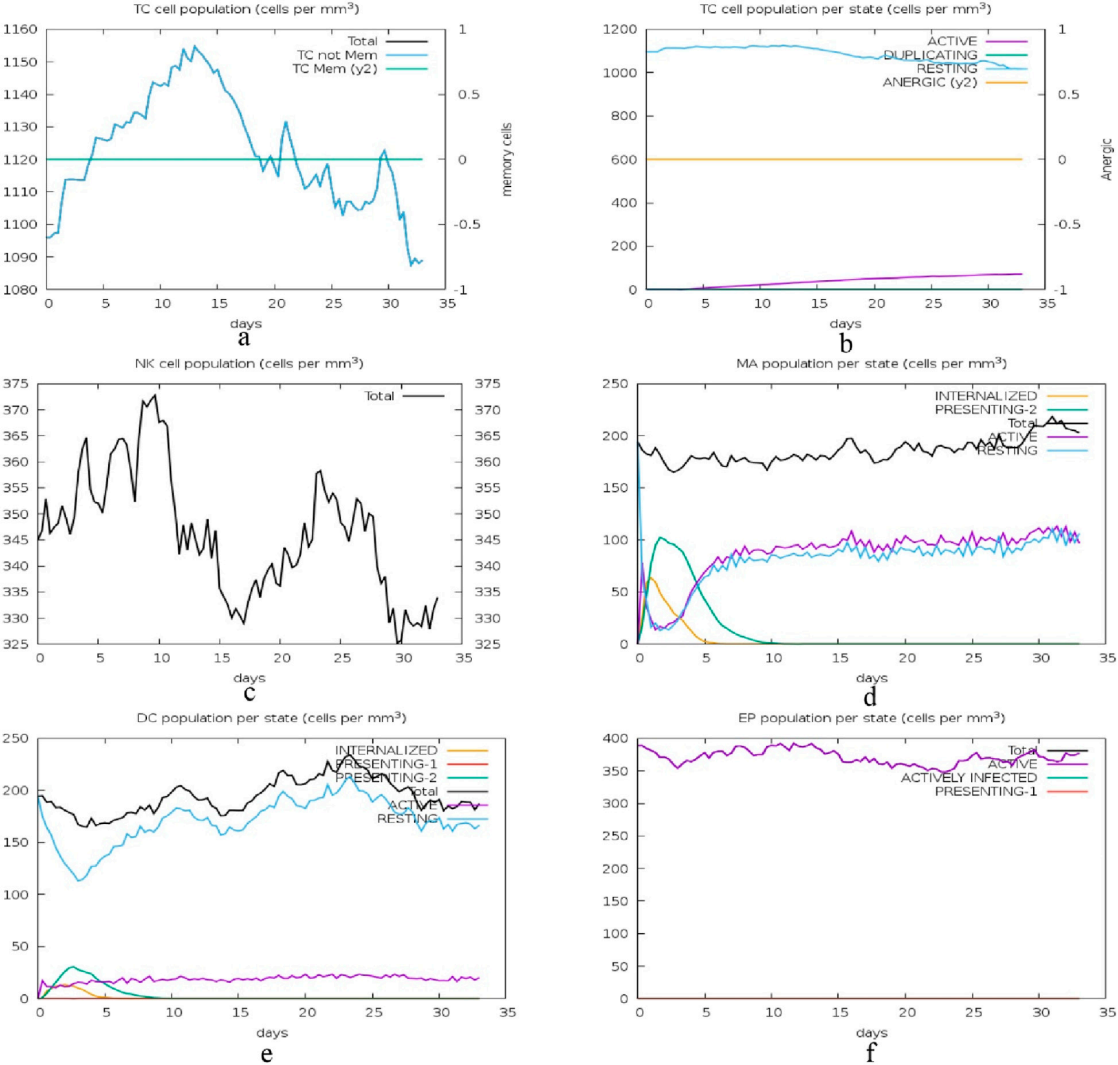
Immune cell state dynamics: this figure illustrates the changes in the immune cell populations and their functional status over a period of 35 days with the aim of depicting the immune surveillance, antigen presentation, and response to infection by cytotoxic T cells (TC), natural killer (NK) cells, macrophages (MA), dendritic cells (DC), and epithelial cells (EP). **(A)** Total cytotoxic T (TC) cell population and memory cells, showing a relatively constant memory cell population, **(B)** TC cell states, highlighting the majority as active or resting, with minimal duplication or anergic cells, **(C)** Natural killer (NK) cell populations, demonstrating variability due to immune monitoring and activity, **(D)** Macrophage (MA) populations, with early increases in internalized and presenting states that level off, reflecting antigen processing and presentation, **(E)** Dendritic cell (DC) states, showing initial increases in internalized cells and stabilization of presenting cells, indicative of antigen presentation functions, and **(F)** Epithelial (EP) cell states, with stable populations of both active and actively infected cells, signifying a balance between infection and immune response.

The second set of graphs presented in Figure 15 depict the kinetics of different immune cell subsets and their performance status. In graph (a), the total cytotoxic T (TC) cell population and its memory cells vary, with memory cells being more constant. Figure b represents the TC cell states, with the majority of the cells being either active or resting with very few cells in the process of duplication or anergic cells. The graph (c) shows the natural killer (NK) cell populations; there is a clear variation in the levels, which may be attributed to the constant immune monitoring and activity. Graph (d) is about the Macrophages (MA) populations and the internalized and presenting states are depicted to rise early and then level off, suggesting antigen processing and presentation. Graph (e) shows the DC states; internalized cells increase initially, and presenting cells stabilize later, indicating their function in antigen presentation. Graph (f) illustrates epithelial (EP) cell states, where both active and actively infected cells are present and stable, meaning that the balance between the infection and immune response is preserved.

**FIGURE 16:**
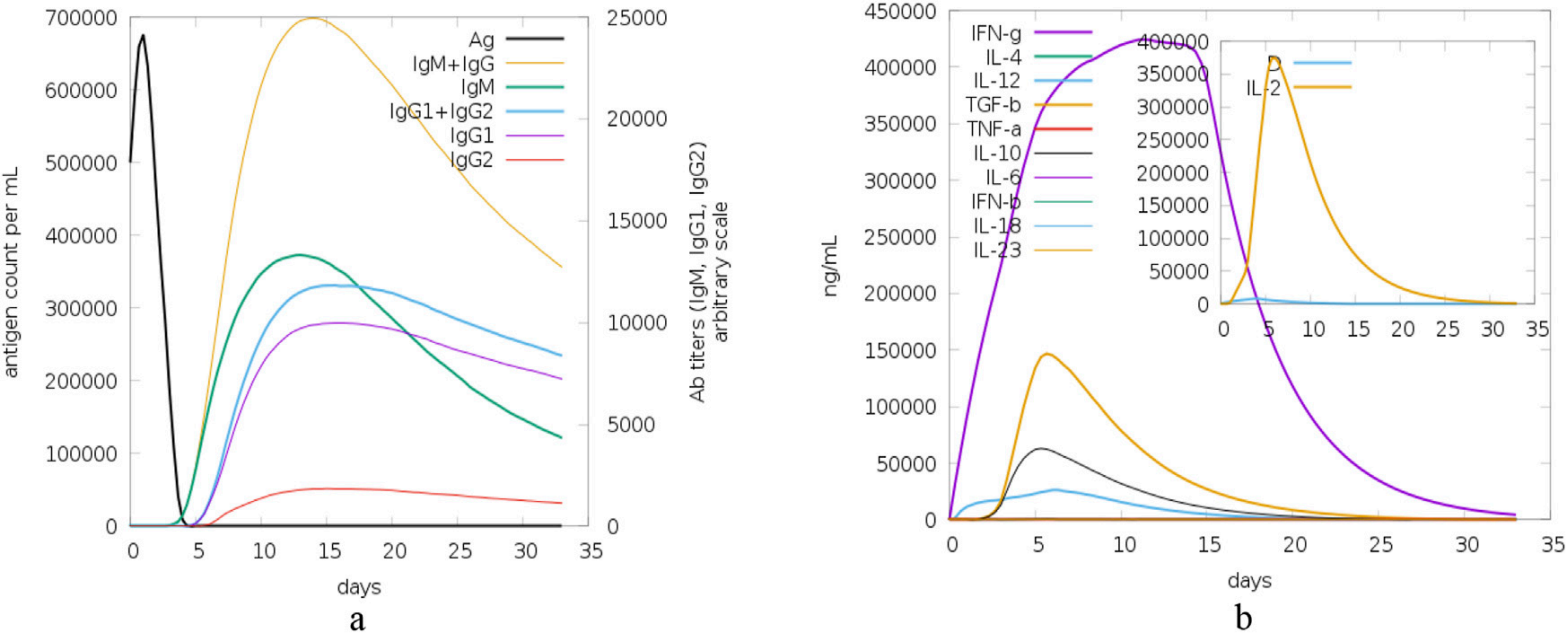
Antigen, antibody and cytokine dynamics: this figure shows the time course of antigen levels, antibody titers and cytokine concentrations during the 35-day study period in order to show the kinetics of the immune response and its regulation. **(A)** The time course of antigen count (Ag) per mL demonstrates a sharp rise followed by a steep decline, indicative of efficient antigen clearance by the immune system. Concurrently, antibody levels for different isotypes (IgM, IgG1, IgG2) exhibit a characteristic pattern, with IgM peaking early and IgG1+IgG2 sustaining elevated levels, reflecting a robust humoral immune response, and **(B)** Time-course profiles of cytokine concentrations, including IFN-γ, IL-4, IL-12, TGF-β, TNF-α, IL-10, IL-6, IFN-β, IL-18, and IL-23. IFN-γ peaks highest, suggesting a dominant Th1 response, while early peaks in cytokines like IL-2 highlight the initial immune response phase.

The last set of graphs presented in Figure 16 depicts the time course of antigen levels and antibody (Ab) titers, and cytokine concentrations. In graph (a), the antigen count (Ag) per mL has a steep rise in the first few hours and then a steep fall which shows that the immune system is clearing the antigen effectively. Thus, the levels of antibodies of different isotypes (IgM, IgG1, IgG2) increase, with IgM reaching the highest level early and IgG1+IgG2 sustaining the higher levels for a longer period, which indicates a strong humoral response. In graph (b), the cytokine concentrations including IFN-γ, IL-4, IL-12, TGF-β, TNF-α, IL-10, IL-6, IFN-β, IL-18, and IL-23 have different time courses. IFN-γ has the highest peak, which indicates that the Th1 response is dominant, while other cytokines such as IL-2 has early and rapid peak which may be related to the early immune response.

### 3.8 Peptide simulation in water

**FIGURE 17:**
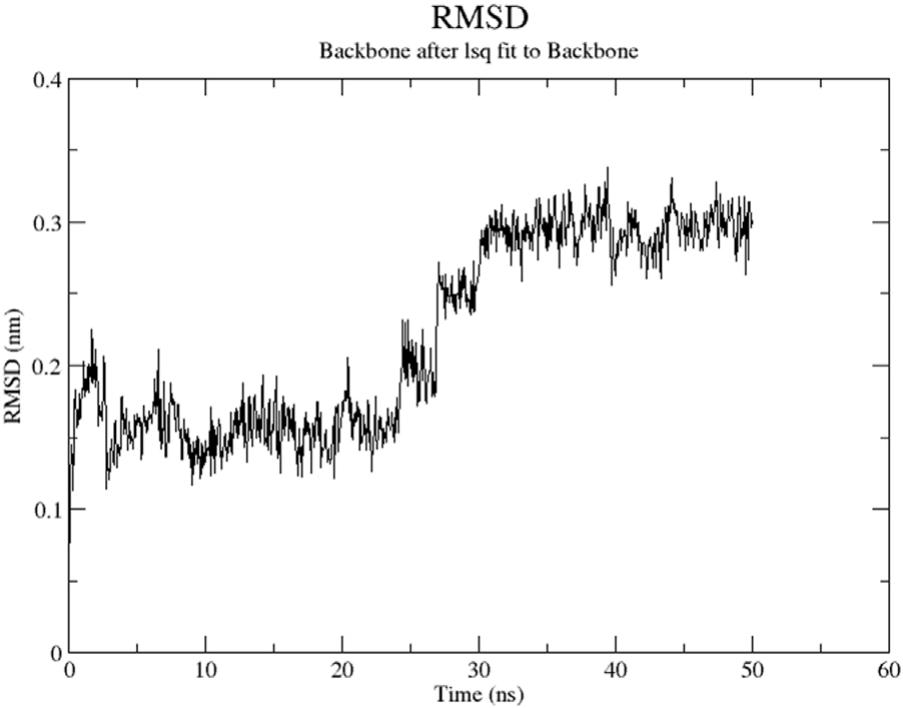
The RMSD graph presents the backbone geometric discrepancies of the peptide in the course of a 50 ns simulation.

**FIGURE 18:**
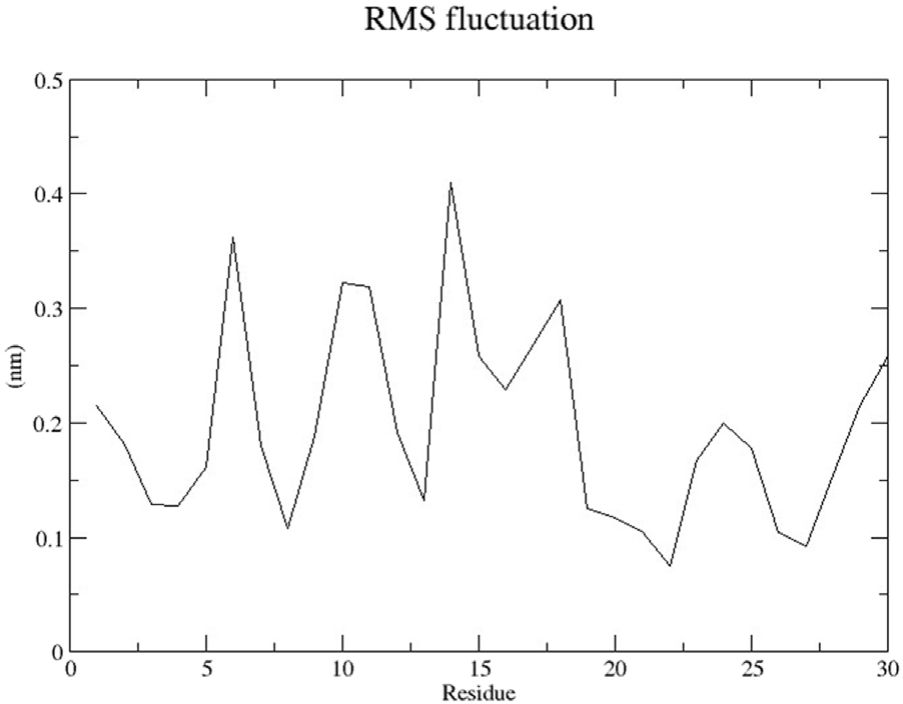
The RMSF graph shows the changes in position of each residue of the peptide during the simulation.

**FIGURE 19:**
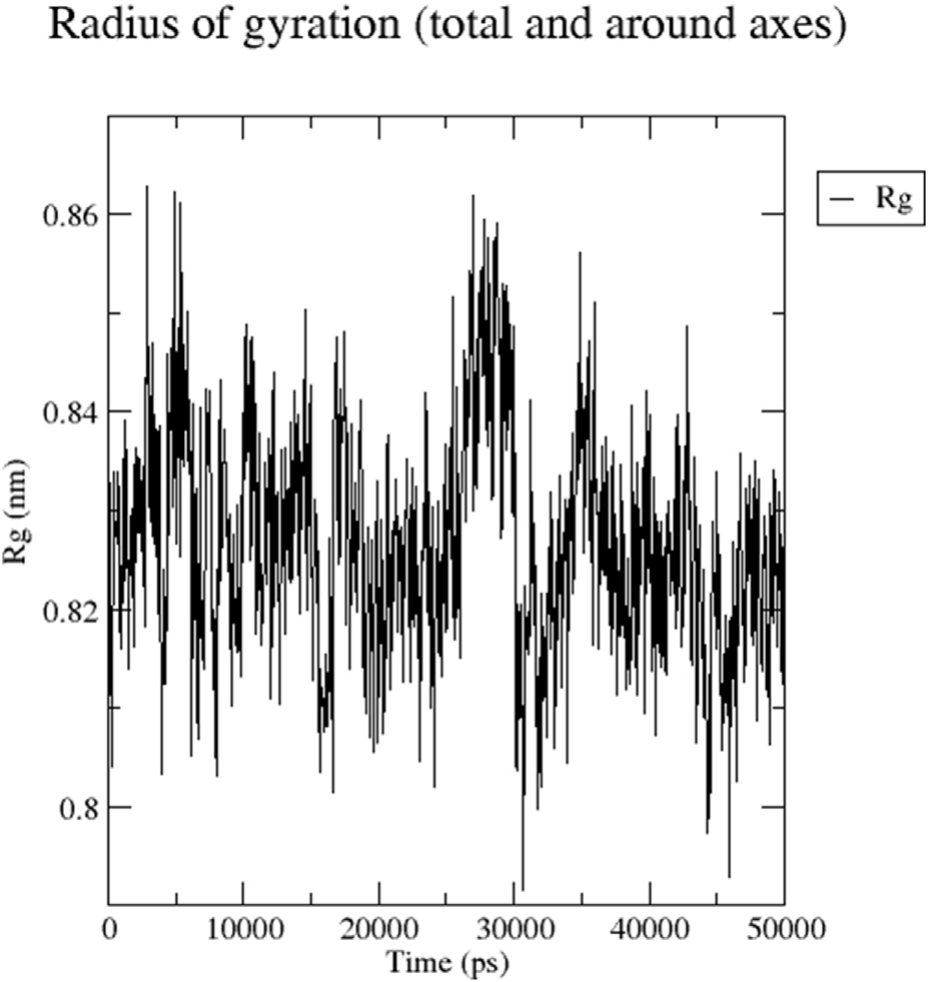
The graph presents the radius of gyration (Rg) of a peptide in water during the time of simulation of 50 ns.

**FIGURE 20:**
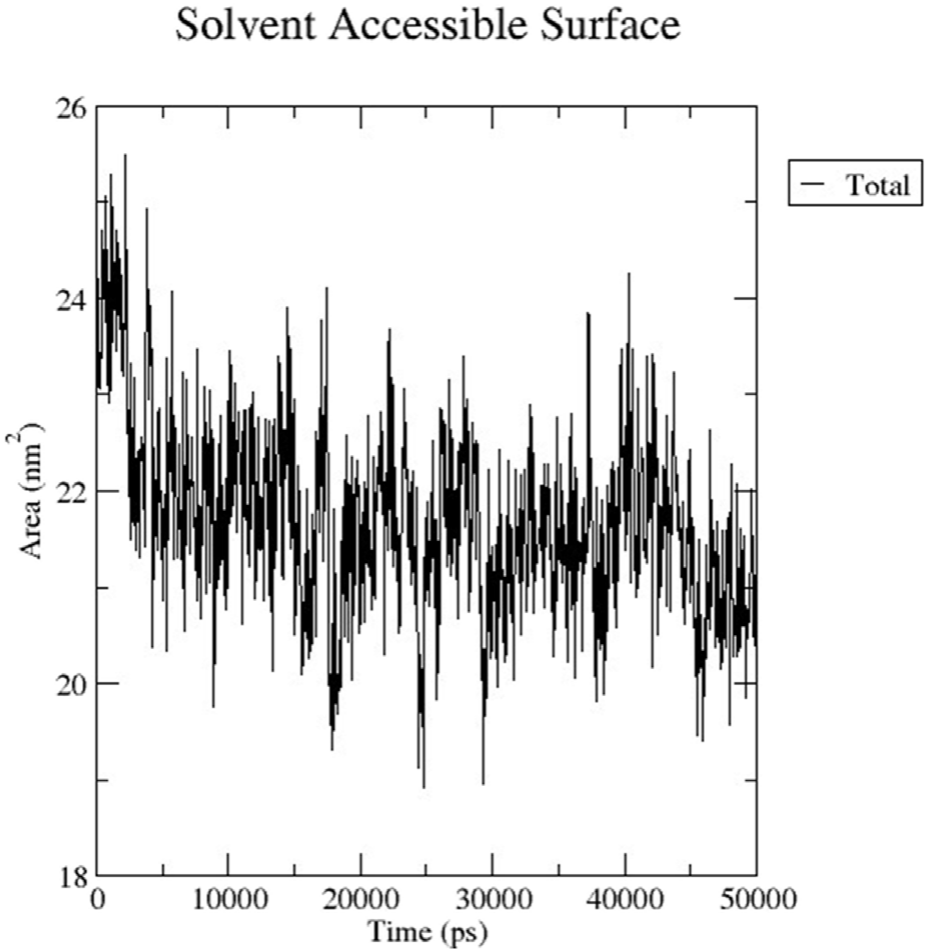
The graph shows the SASA of the peptide for the 50ns of the simulation.

**FIGURE 21:**
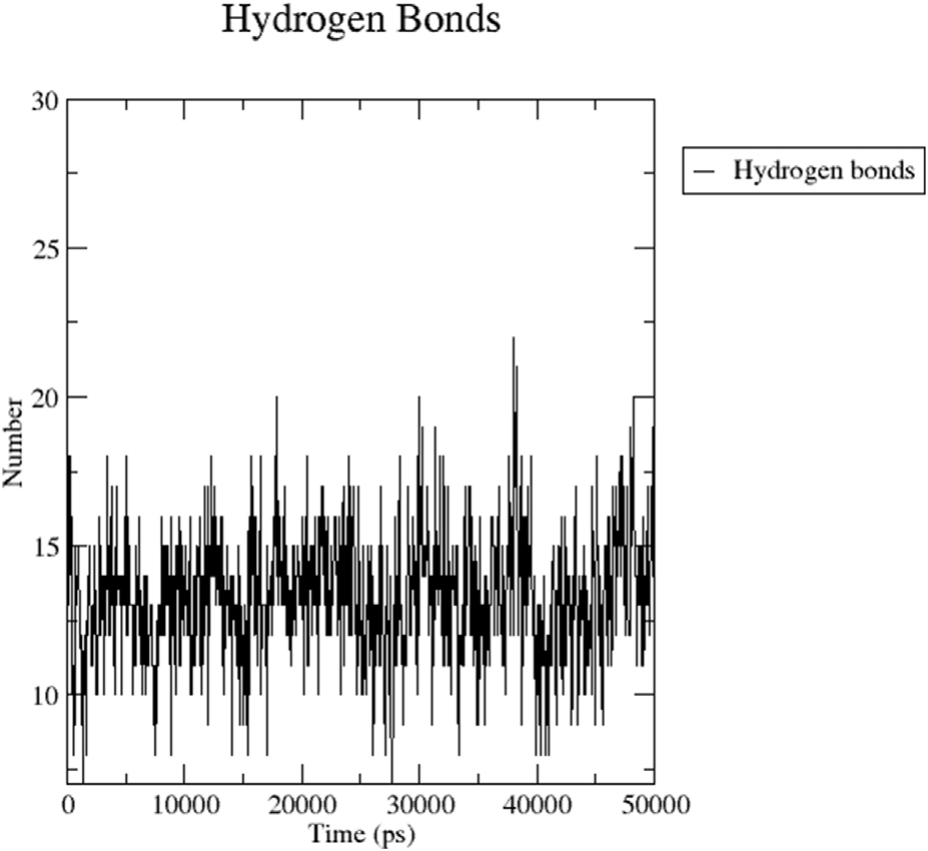
The graph shows the number of hydrogen bonds in a peptide at the course of 50 ns simulation.

The RMSD graph presented in Figure 17 depicts the backbone atomic deviations of the peptide during a 50 ns molecular dynamics simulation. It shows structural stability with the RMSD fluctuating around 0.15–0.2 nm during the first 25 ns The RMSD rises to 0.3 nm after 25 ns, indicating a conformational change. It then stabilizes onto another equilibrium state around 0.3 nm between 30 and 50 ns RMSF of the Globa D peptide is depicted in Figure 18. Fluctuations of 0.4 nm observed on residues surrounding 6, 12, and 16 nm may enhance structural adaptability or facilitate binding interactions. In contrast, fluctuations are low, with residues between 20 and 25 staying around 0.15 nm or less, which shows a stable core region needed to support the structure of the peptide. Figure 19 presents the radius of gyration of a peptide in water as a function of the simulation time, which is equal to 50 ns The increase in the gyration (Rg) radius for the Globa D peptide over time represents how stable the peptide remains during the simulation. The value of the Rg mostly remains below 0.84 nm, pointing to peptide compact conformation. The reversible fluctuations of 0.8–0.86 nm suggest dynamic structural changes within a stable overall structure. Figure 20 plot shows the solvent-accessible surface area (SASA) which measures the amount of the peptide in contact with the solvent during the simulation of the peptide during the course of the 50 ns of the simulation. Initially, the fluctuation of SASA is around 23–24 nm^2^ and eventually stabilizes in the range between 19 nm^2^ and 22 nm^2^. The graph presented in Figure 21 shows of the number of hydrogen bonds in the Globa D peptide over time to determine the stability of the structure along the course of the simulation, as well as any internal interactions in the structure. Over the simulation period of the trajectory, the fluctuating number of hydrogen bonds ranges from 10 to 20 bonds, with occasional peaks as high as approximately between 20 and 25. These fluctuations suggest that the peptide has structural integrity with a hydrogen-bonded network of the peptide without losing stability.

## 4 Discussion

In current research, the PorB porin protein of *Neisseria gonorrhoeae* was obtained from the Protein Data Bank (PDB) with the ID 4AUI. The protein structure was cleaned and visualized using Discovery Visual Studio (Biovia). It possesses chains: Chain A, B, and C. The protein secondary structure predicted by PSIPRED is mainly composed of beta strands, typical of the beta-barrel, which is required to form the pore in the bacterial membrane. The ProtParam tool on the ExPASy server was used to predict the protein’s physicochemical properties. It found that the protein has no net charge; however, it has a feeble positive charge as there are 38 positively charged amino acids and 32 negatively charged ones. The extinction coefficient of 54,320 M-1 cm-1 at 280 nm is high, showing high absorbance, typical of aromatic amino acids. PorB has a low instability index of 30.72 and a moderate aliphatic index of 67.40, which helps maintain the stability of the protein. The negative GRAVY score is −0,539, which means that the protein is hydrophilic (Babnigg and Joachimiak, 2010). The value of the protein’s calculated theoretical isoelectric point (pI) is 9.14, which indicates that the protein is basic and can affect its properties and solubility based on pH. At pH levels below 9.14, the protein is expected to carry a net positive charge, leading to decreased solubility and potential precipitation (Rothstein, 2019).

Docking analysis was performed using the HDOCK tool to predict the interaction between the PorB porin and potential cyclotides (Raghav et al., 2019). Globa D was chosen as the best drug candidate because it represented the complex with the top docking score (−270.04) among all the complexes yielded by docking calculations. Interaction analysis revealed that the docking complex have 7 hydrogen bonding interactions between amino acids of receptor and ligand revealing a strong binding affinity. Globa D is a small cyclic protein isolated from the plant Gloeospermum blakeanum. It has a particular order of amino acids and was characterized by PCR methods. Globa D is a wild-type protein, which means that it is not genetically altered in any way. However, specific tests (assays) have yet to be conducted on this protein, and the information has been obtained from a study conducted by Burman, Gruber (Burman et al., 2010). Globa D has an SVM score of −0. 37 is considered safe, does not cause allergic reactions, and is non-toxic. This property of allergenicity and toxicity was predicted using AlgPred and ToxinPred, respectively (Lafarga et al., 2016). According to its physiochemical properties predicted by using ProtParam, it consists of 30 amino acids and 428 atoms, and the molecular weight of this protein is 3,224.90. This protein has a low amount of charged amino acids, and the extinction coefficients of the protein differ depending on the oxidation state of cysteine residues. Globa D has moderate stability with an Aliphatic Index of 45.33, and the instability index stands at 17. 30. The GRAVY score of 0.410 suggests moderate hydrophobicity, and the theoretical pI of the protein is 7.79, which shows that it is slightly basic (Mehta et al., 2022).

MD simulations were performed for 100,000 picoseconds using Desmond from Schrödinger LLC (Chinnasamy et al., 2020b). The principal component analysis (PCA) and dynamic cross-correlation matrix (DCCM) were also evaluated by applying the R package “Bio3D” (Raghavan et al., 2022). The MM-GBSA analysis was performed using the OPLS 2005 parameters in Prime (Schrödinger Suite) (Du et al., 2011). The findings indicate structural flexibility in some regions of PorB Porin, which might be crucial for its biological activity or interaction with ligands. The structural stability of Globa D is further supported by the low values of RMSD and RMSF and good binding interaction with PorB Porin. The value of the radius of gyration demonstrates that PorB Porin stays in an extended conformation, while the compact structure of Globa D ensures its stable interaction with the receptor. The changes in the hydrogen bonds mean that the binding interaction is not in an equilibrium state; the binding affinities are different at different time points in the simulation, which may suggest that the ligand-receptor interaction is flexible. Considered favorable, and it is understood that the ligand has moderate binding to the target (Cournia et al., 2017).

The immune response to a cyclotide-targeted protein complex was studied by simulating its interactions using the C-ImmSim 10.1 server (Chatterjee et al., 2021). The results, assessed using the PSSM technique, demonstrate the robustness and effectiveness of the immune system’s response. The time course of the changes in the number of different immune cells reveals a well-coordinated response. B cells, including memory cells and various isotypes (IgM, IgG1, IgG2), show a rise and then level off, with plasma cells producing IgM first and in the highest amount. TH cells, both total, and memory, also show a sharp rise and then plateau, moving from the activated to the resting or anergic state. The TH cell subtypes (Th0, Th1, Th2, Th17, Treg) also grow and become more stable. Cytotoxic T (TC) cells and their memory subsets differ; memory cells are more stable while TC cells are active or resting. Natural killer (NK) cells have different levels, indicating they are constantly active. Macrophages (MA) and dendritic cells (DC) increase early, suggesting antigen presentation. Epithelial (EP) cells are in a constant state of activation and infection. Antigen levels increase and then decrease, suggesting that the immune system has effectively cleared the pathogen, while antibodies increase early (IgM) and are sustained (IgG1, IgG2), suggesting a robust humoral response. Cytokine levels are different, but IFN-γ is the highest, which shows that the Th1 response is dominant, and other cytokines, such as IL-2, are early peaked, which shows the initial immune response. Overall, these simulations give us a conception of the immune system’s perfect response to an antigen, which means that the immune system recognizes the antigen, initiates a proper response against it, and eradicates it.

Globa D Simulation in water on 50ns results shows the ability of the peptide for aqueous-based drug formulation. Analysis of the RMSD shows that the peptide is structurally stable over time, with only minimal conformational change, indicating that the peptide can maintain its functional structure in water. The RMSF results indicate a balance of flexible and stable residues with a few regions with high mobility that could help facilitate effective binding to the porin of *N. gonorrhoeae*. While allowing for some dynamic flexibility required for biological interactions, the peptide’s radius of gyration stays in a narrow range, suggesting that the peptide is compact. SASA analysis also reveals that solvent exposure decreases gradually, leading to stable conformation of the peptide in water, minimizing the risk of rapid degradation. Last, consistent hydrogen bonds throughout the simulation indicate the internal stability required for the peptide to retain its integrity in an aqueous environment. These results imply that the peptide is a good candidate for water-soluble drug formulation, balancing structural rigidity, flexibility, and functionality necessary to interact with the target.

The research conducted byAli (2022) includes molecular docking using AutoDock Vina, molecular modelling using PyMOL, and toxicity prediction using SWISS-ADME to identify potential inhibitors against antibiotic-resistant *Neisseria gonorrhoeae* from natural marine fungus-derived compounds. Elipyrone A has a binding affinity of −8.5 kcal/mol and is found to be highly drug-like in terms of solubility and pharmacokinetic properties. The current study involves the PorB Porin protein and cyclotides; HDOCK is used for docking while the protein structure is visualized in Discovery Visual Studio; the interaction of the two is viewed in PyMOL; ToxinPred and AlgPred are used to predict toxicity and allergenicity, respectively. Globa D has the highest docking score of −270.04, and the structural stability and safety in the simulations are confirmed by molecular dynamics and MM-GBSA energy prediction, which gives information about the binding stability and conformational changes in time. The current study also uses immune simulations in the C-ImmSim server to predict the immune response and peptide simulation in water to test the solubility and stability of the compound, which gives a better assessment of the therapeutic value. Although both studies are free from methodological drawbacks, the current study employs more tools and simulations and can be viewed as more comprehensive and prospective. The implications point to two potential sources of novel drugs, the marine-derived compounds and the plant-derived cyclotides against the MDR *N. gonorrhoea*, crucial for the development of new antibiotics to deal with this emerging health threat with the approaches used in the current study, possibly offering a more efficient way of identifying new drugs.

## 5 Limitations and future directions

The current study effectively shows the possibility of cyclotides as drug for *Neisseria gonorrhoeae* PorB Porin protein, this work is mainly computational, which is strong but only in the initial phase of drug discovery. Future directions should involve further *in vitro* and *in vivo* studies to confirm the effectiveness and the toxicity of Globa D. Furthermore, if the structure of Globa D is to be altered, its efficiency as a therapeutic compound could be improved. Extending this research to pharmacokinetic and pharmacodynamic investigations is also necessary for further development of these observations for clinical uses.

## 6 Conclusion

Globa D proved the best drug candidate against *Neisseria gonorrhoeae* by inhibiting chain A of PorB Porin protein with the top docking score (−270.04). It shows no allergenicity and toxicity. It also showed favorable MD simulations, immune stimulation, and peptide simulation in water results and can be an excellent aqueous drug applications in the clinic.

## Data Availability

The datasets presented in this study can be found in online repositories. The names of the repository/repositories and accession number(s) can be found in the article/Supplementary Material.
